# Autophagy alterations in obesity, type 2 diabetes, and metabolic dysfunction-associated steatotic liver disease: the evidence from human studies

**DOI:** 10.1007/s11739-024-03700-w

**Published:** 2024-07-06

**Authors:** Patrycja Jakubek, Barbara Pakula, Martin Rossmeisl, Paolo Pinton, Alessandro Rimessi, Mariusz Roman Wieckowski

**Affiliations:** 1 Laboratory of Mitochondrial Biology and Metabolism, Nencki Institute of Experimental Biology, Polish Academy of Sciences, 3 Pasteur St., 02-093 Warsaw, Poland; 2 Laboratory of Adipose Tissue Biology, Institute of Physiology of the Czech Academy of Sciences, Prague, Czech Republic; 3 Department of Medical Sciences, Section of Experimental Medicine, Laboratory for Technologies of Advanced Therapies, University of Ferrara, Ferrara, Italy; 4 Center of Research for Innovative Therapies in Cystic Fibrosis, University of Ferrara, 44121 Ferrara, Italy

**Keywords:** Metabolic diseases, Patients, Tissue biopsy, Cellular quality control, Autophagy modulators, Therapies

## Abstract

Autophagy is an evolutionarily conserved process that plays a pivotal role in the maintenance of cellular homeostasis and its impairment has been implicated in the pathogenesis of various metabolic diseases including obesity, type 2 diabetes (T2D), and metabolic dysfunction-associated steatotic liver disease (MASLD). This review synthesizes the current evidence from human studies on autophagy alterations under these metabolic conditions. In obesity, most data point to autophagy upregulation during the initiation phase of autophagosome formation, potentially in response to proinflammatory conditions in the adipose tissue. Autophagosome formation appears to be enhanced under hyperglycemic or insulin-resistant conditions in patients with T2D, possibly acting as a compensatory mechanism to eliminate damaged organelles and proteins. Other studies have proposed that prolonged hyperglycemia and disrupted insulin signaling hinder autophagic flux, resulting in the accumulation of dysfunctional cellular components that can contribute to β-cell dysfunction. Evidence from patients with MASLD supports autophagy inhibition in disease progression. Nevertheless, given the available data, it is difficult to ascertain whether autophagy is enhanced or suppressed in these conditions because the levels of autophagy markers depend on the overall metabolism of specific organs, tissues, experimental conditions, or disease duration. Owing to these constraints, determining whether the observed shifts in autophagic activity precede or result from metabolic diseases remains challenging. Additionally, autophagy-modulating strategies are shortly discussed. To conclude, more studies investigating autophagy impairment are required to gain a more comprehensive understanding of its role in the pathogenesis of obesity, T2D, and MASLD and to unveil novel therapeutic strategies for these conditions.

## Introduction

The increasing prevalence of metabolic diseases is a growing global health concern with significant implications for public health [1–3]. According to global World Health Organization (WHO) estimates, in 2016, 650 million adults worldwide (18 years old and older) were obese, and 340 million children and adolescents (age: 5–19) were either overweight or obese. The global prevalence of obesity nearly tripled between 1975 and 2016, and if this trend continues, even 1 billion adults may develop obesity by 2025 [4]. In line with these estimates, in May 2022, the WHO released a European Regional Obesity Report, which assessed that up to 60% of adults in the European WHO region are either overweight or obese [5]. The rising prevalence of obesity is often accompanied by other comorbidities, such as type 2 diabetes (T2D). At the end of 2021, the International Diabetes Federation accounted for approximately 500 million diabetes mellitus patients worldwide, of which over 90% were diagnosed with T2D. This number is predicted to continue growing in the following years [6]. Furthermore, even 50–70% of T2D patients, as well as 30–76% of obese and up to 90% of morbidly obese individuals, can also be affected by metabolic dysfunction-associated steatotic liver disease (MASLD), formerly known as non-alcoholic fatty liver disease (NAFLD) [7, 8], which in recent years became the most common chronic liver disease worldwide [1]. Importantly, there is a direct relationship between these three conditions, as the existence of one condition increases the risk of the other. Their coexistence is related to, among others, common risk factors (e.g., insulin resistance and metabolic syndrome), some of which may be modifiable (e.g., physical inactivity or unhealthy diet).

Unsurprisingly, the pathogenesis of obesity, T2D, and MASLD shares common mechanisms such as mitochondrial dysfunction or oxidative and endoplasmic reticulum (ER) stress, chronic inflammation, gut dysbiosis, and altered autophagy [9, 10]. Autophagy is a key evolutionarily conserved cellular process essential for degrading and recycling damaged or malfunctioning cellular components, which can be reused for biosynthetic purposes or energy generation [11]. As such, at basal levels, autophagy contributes to maintaining cellular homeostasis, survival, and various aspects of metabolic health [12]. Autophagy has been intensively studied since its discovery in 1963 [13] and is still a subject of growing interest among researchers and clinicians [14]. Its regulation by an array of signaling pathways depends on nutrient and energy availability, thus it is inherently related to the metabolism of amino acids, glucose, and lipids. Understanding these interrelations is crucial for elucidating the complex mechanisms underlying a variety of diseases, including genetically inherited metabolic disorders, such as lysosomal storage disorders, as well as non-communicable diseases (e.g., obesity, T2D, and MASLD). This review aims to provide an overview of the clinical evidence regarding the role of autophagy impairments in the development of obesity, T2D, and MASLD. Describing detailed molecular mechanisms is outside the scope of this review; however, for more details, the reader can refer to [15–17].

## Autophagy and its regulation

Autophagy, as a process of self-degradation, plays a significant role in maintaining cellular homeostasis. It is active under basal conditions and can be further stimulated in response to cellular stress such as starvation, enabling the cell to mobilize essential energy sources through the degradation of glycogen (storage of glucose), lipid droplets (storage of triacylglycerols), or other intracellular structures [18]. The physiological regulation of autophagy has been elegantly reviewed in a paper by Rabinowitz and White so the reader can refer to this work for more information [11]. Autophagy also serves as a protective mechanism by eliminating damaged organelles or misfolded proteins that can exert cytotoxic effects [16, 19].

In mammalian cells, three types of autophagy can be identified: microautophagy, macroautophagy, and chaperone-mediated autophagy (CMA) [20]. Despite morphological differences, all these processes culminate in delivering cargo to the lysosome for degradation and recycling. Microautophagy relies on the invagination or protrusions of the lysosomal membrane to capture cargo. CMA utilizes chaperones to identify the proteins targeted for degradation. Meanwhile, macroautophagy, to which in this review, we refer simply as "autophagy", involves isolation of the cargo by forming a double-membraned structure called an autophagosome, which subsequently fuses with a lysosome and its content undergoes degradation [18]. Furthermore, autophagy can be either non-selective or selective [16]. Non-selective macroautophagy plays a crucial role during starvation, involving the random engulfment of cytoplasmic fragments into autophagosomes. Subsequently, lysosome fusion with the autophagosome provides luminal acid hydrolases that degrade captured proteins, lipids, carbohydrates, nucleic acids, and organelles. Such degraded material supplies nutrients that are then secreted back into the cytoplasm by lysosomal permeases to provide the cell with essential energy sources under conditions of cellular stress [21]. Conversely, selective macroautophagy specifically recognizes and degrades a defined cargo, such as protein aggregates (aggrephagy), organelles (e.g., mitophagy, lysophagy, ER-phagy, ribophagy, lipophagy, pexophagy), and pathogens (xenophagy) [16]. Hence, selective autophagy plays a significant role in cellular quality control. Despite the common mechanisms involved in organelle removal, the degradation signals and molecules used in selective autophagy are diverse and specific to each organelle. A comprehensive understanding of these pathways is crucial because defects in the selective autophagy of various organelles have been associated with conditions such as metabolic disorders, neurodegeneration, and aging [16]. Detailed knowledge of how cargos are recognized is necessary for developing specific therapies that precisely target individual stages of autophagy in clinical practice [22].

**Fig. 1:**
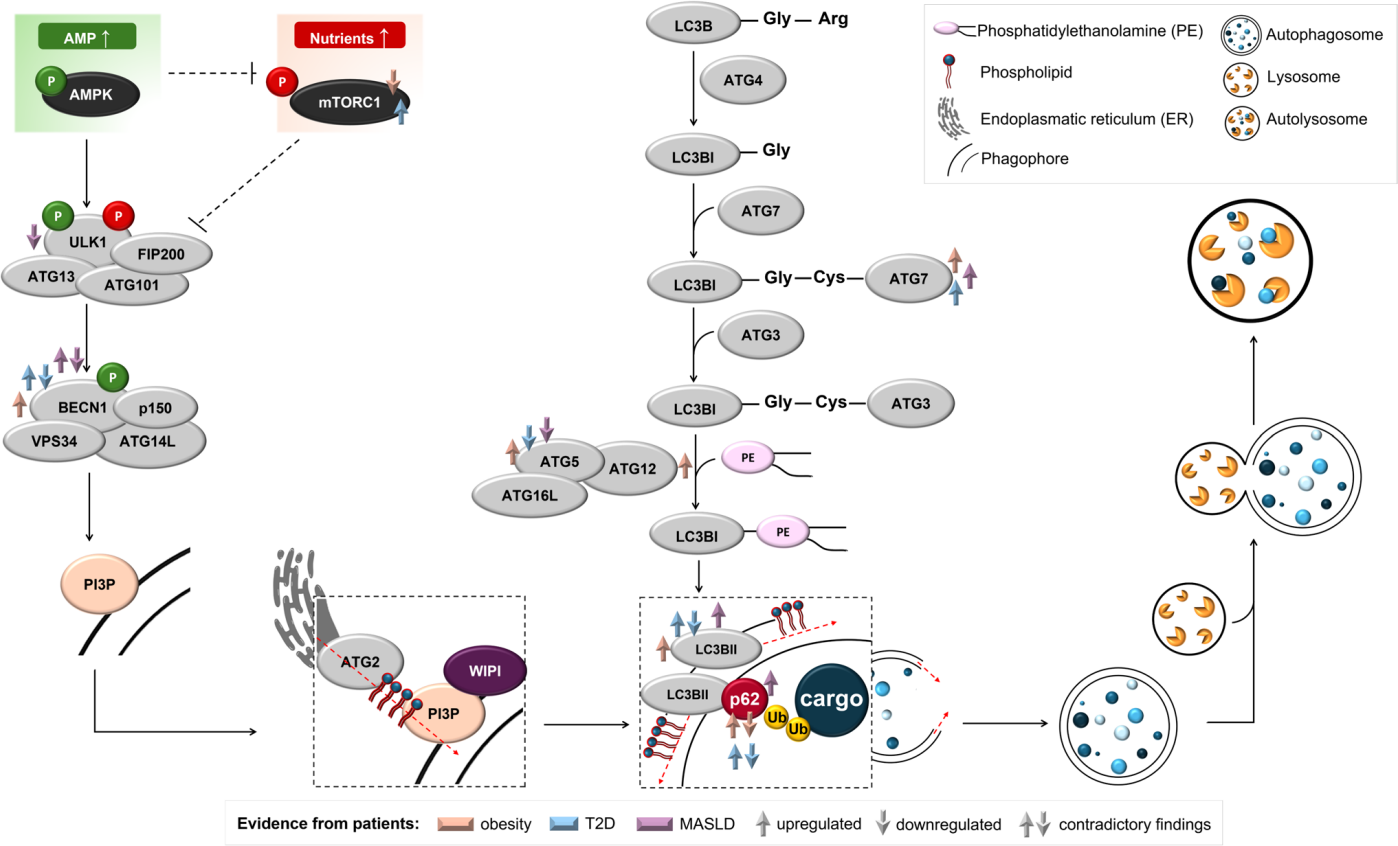
The primary mechanism initiating autophagy involves activation of the Unc-51-like kinase (ULK) complex, consisting of ULK1/ATG1, ATG13, FIP200, and ATG101. The key regulators of autophagy initiation are the mTORC1 complex and AMP-activated protein kinase (AMPK), which act in opposition to each other; however, both control autophagy through ULK1 phosphorylation. AMPK, the primary sensor of cellular energy state, is activated when intracellular AMP levels rise (indicating starvation) and then promotes autophagy by directly activating ULK1 through the phosphorylation of Ser317 and Ser777. Under conditions of nutrient sufficiency, mTORC1 prevents ULK1 activation by phosphorylating Ser757 on ULK1 and disrupting the interaction between ULK1 and AMPK [30, 31]. The ULK1 complex further activates the BECN1-VPS34-ATG14L-p150 complex through the phosphorylation of Beclin 1 (BECN1). Activation of the BECN1 complex leads to the generation of phosphatidylinositol-3-phosphate (PI3P), which is crucial for the nucleation of autophagic vesicles by promoting membrane elongation through the recruitment of the ATG2-WIPI (WD‐repeat protein interacting with phosphoinositides) protein complex. The elongation and maturation of autophagosomes involve two conjugation systems similar to the ubiquitination system: the microtubule-associated protein 1 light chain 3 (LC3/ATG8) system and the ATG12 system [32]. LC3 is modified by ATG4, resulting in LC3-I with an exposed glycine residue at the C-terminus. This allows the conjugation of LC3-I with ATG7 (an E1-like enzyme) and then with ATG3 (an E2-like enzyme) [23]. ATG3-LC3 is recognized by the ATG5-ATG12 complex associated with the ATG16L protein (ATG16L complex), which catalyzes the conjugation of LC3 with phosphatidylethanolamine (PE), forming insoluble LC3-II that is stably incorporated into the autophagosomal membrane [33, 34]. Interestingly, cargo selection for the autophagy process can be facilitated by adaptor proteins, such as p62/SQSTM1, which possess a ubiquitin-binding domain and an LC3-II interacting domain. The fusion of autophagosomes and lysosomes is regulated by several molecules including soluble N-ethylmaleimide-sensitive factor attachment protein receptors (SNAREs) and lysosome-associated membrane proteins (LAMPs) [21, 32]. Finally, in the last step of autophagy, the encapsulated cargo is degraded by lysosomal proteases, and the products are released back into the cytosol through lysosomal permeases [32]

In most cases, autophagy consists of five steps: initiation, nucleation of the autophagosome, expansion, and elongation of the autophagosome membrane, closure and fusion with the lysosome, as well as degradation of intravesicular products [23]. Autophagy is controlled by over 30 autophagy-related proteins encoded by autophagy-related genes (ATGs), originally identified in the yeast *Saccharomyces cerevisiae* [21]. A crucial regulator of autophagy activation is the mammalian target of rapamycin (mTOR) kinase, which acts as the central component of two functionally and structurally distinct complexes: mTORC1 and mTORC2. mTORC1 activity is inhibited in response to amino acid starvation, growth factor deprivation, decreased ATP or oxygen levels, and enhanced reactive oxygen species (ROS) production. As such, mTORC1 inhibition promotes the initiation of autophagy [24]. The role of mTORC2 in autophagy is not well understood; however, studies suggest that this complex may promote both the activation [25, 26] and inhibition of autophagy [27–29]. The process from autophagy initiation to cargo degradation is illustrated in Fig. 1.

## Autophagy in metabolic diseases

The pathological mechanisms that drive the development of obesity, T2D, and MASLD include a combination of predisposing genetic background and accompanying environmental and behavioral factors, such as excessive caloric intake and lack of physical activity [35]. Despite their impact on, among others, systemic hormonal and metabolic regulation, these factors are also known to either enhance or suppress autophagy. Autophagy alterations can further affect both adipogenesis and metabolism at the tissue level, potentially manifesting observable effects at the systemic level [36]. Considering the complex and multifactorial pathogenesis of metabolic diseases, in this review, we discuss autophagy alterations in obese patients in the context of T2D and MASLD, as many obese patients commonly suffer from these comorbidities [37].

### Obesity

The first observations of changes in autophagy in obese patients were reported in 2010 by Öst et al., who with the use of transmission electron microscopy (TEM) reported an increased number of autophagosomes in adipocytes obtained from obese patients with T2D [38]. This increase occurred together with enhanced levels and turnover of the microtubule-associated protein 1 light chain 3 (LC3) protein, one of the main autophagy markers, as demonstrated in the presence and absence of an autophagy inducer (rapamycin) and inhibitor of lysosomal degradation (chloroquine). The reduced activity of mTORC1 further supported these findings. Furthermore, the observed stimulation of autophagy was associated with the fragmentation of large lipid droplets. In the studied cohort, the average BMI of patients with T2D was approximately 40, pointing to obesity class II/III; however, overweight subjects (BMI ≥ 27) were also included. Patients without T2D included in this study as controls were overweight on average [38]. However, Kovsan et al. showed that autophagophore formation was upregulated in adipose tissue (AT) obtained from obese patients, regardless of their glycemic status. This effect was particularly pronounced in the omental compared to the subcutaneous fat depot, as observed at both mRNA and protein levels (Table 1). This upregulation was positively correlated with the degree of obesity, visceral fat distribution, and adipocyte hypertrophy [39]. In the following years, more studies reported similar findings of upregulation of autophagy markers in AT from patients with obesity and obesity-related T2D, compared to lean controls without T2D [40, 41]. Strong positive correlations between the expression levels of autophagy-related genes and glucometabolic status [42], or higher transcript levels of autophagic genes in the AT of patients with obesity and T2D compared to those with normal glycemia, have also been reported [43]. The increase in autophagosome formation was also shown to be more pronounced in the visceral AT than in the subcutaneous AT [38, 39]. In contrast to patients with obesity and T2D, LC3 was not detected in subcutaneous and visceral AT samples from lean individuals without T2D [41]. Accordingly, as shown in another study, an initially increased number of autophagosomes in the subcutaneous AT of obese patients with or without T2D was undetectable 1 year after they were subjected to bariatric surgery [40]. It has been suggested that stimulated autophagy in AT of individuals with obesity probably plays a role in the modulation of obesity-induced AT inflammation. This assumption is supported by the findings that the inhibition of autophagy significantly enhanced the transcriptional expression of proinflammatory cytokines in obese human and murine AT samples [44].

**Table 1:** Studies demonstrating autophagy alterations in patients with obesity

Cohort	Materials	Autophagy markers	Interpretation	References
On average overweight, controls without T2D^a^ Obese/overweight patients with T2D	Adipocytes isolated from subcutaneous AT	Increased number of autophagosomes Increased level and turnover of LC3 protein in the presence of lysosomal degradation inhibitor	Upregulated autophagosome formation	[38]
Cohort 1: obese and non-obese individuals (*n* = 65) Cohort 2: lean, subcutaneous obese, and intra-abdominally obese individuals (*n* = 192) Cohort 3: severely obese individuals without T2D or with obesity-associated morbidity, matched for being insulin-sensitive or resistant (*n* = 60)	Omental and subcutaneous AT	Increased mRNA and protein levels of *ATG5, LC3A, LC3B* in: - Omental compared to subcutaneous AT - Obese individuals, particularly with intraabdominal adiposity No significant alterations in autophagy markers were found in patients with T2D compared to those without T2D	Upregulated expression of genes involved in autophagosome formation	[39]
Lean controls^b^ Obese normoglycemic individuals Obese individuals with impaired glucose tolerance Obese individuals with T2D	Stromal-vascular fraction cells (SVFC) and adipocytes isolated from omental AT	Increased mRNA levels of *ATG7* and *BECN1* in omental white AT of obese patients with T2D compared with lean controls and normoglycemic obese individuals There were no differences in mRNA levels of *ATG5, ATG7*, and *BECN1* between SVFCs and adipocytes	Upregulated expression of genes involved in autophagosome formation	[43]
Lean controls (*n* = 17) Obese individuals (*n* = 16)	Subcutaneous AT	Increased level of LC3-II protein	Upregulated autophagosome formation	[44]
Lean controls without T2D (*n* = 8) Obese individuals without T2D (*n* = 9) Obese individuals with T2D (*n* = 6)	Subcutaneous AT	Increased number of autophagosomes and BECN1 protein level in AT from obese patients with and without T2D Both markers significantly dropped after body mass reduction (1 year after bariatric surgery)	Increased autophagosome formation	[40]
Lean controls without T2D (*n* = 20) Obese individuals (*n* = 20) Obese individuals with T2D (*n* = 20)	Visceral and subcutaneous AT	Increased LC3-II/LC3-I protein ratio Up-regulated *LC3* and *ATG5* mRNA levels Decreased levels of p62/SQSTM1 and mTOR proteins	Upregulated autophagosome formation and decreased lysosomal degradation	[41]
Nonobese controls (*n* = 12)^c^ Obese individuals (*n* = 24)	Adipocytes isolated from subcutaneous AT	Increased mRNA and protein level of *p62/SQSTM1* Decreased autophagic flux (LC3-II protein accumulation)	Decreased lysosomal degradation	[45]
**Leipzig cohort:** Lean controls (*n* = 102) Overweight individuals (*n* = 67) Obese individuals (*n* = 268) **Beer-Sheva cohort:** Lean controls (*n* = 14) Overweight individuals (*n* = 11) Obese individuals (*n* = 44)	Omental and subcutaneous AT	Increased mRNA and protein levels of *ATG5, and LC3B* in omental AT	Upregulated autophagosome formation	[47]
Lean controls without T2D (*n* = 9) Overweight/obese individuals with impaired glucose tolerance (*n* = 9) Overweight/obese individuals without impaired glucose tolerance (*n* = 8)	Subcutaneous AT Differentiated human multipotent adipose-derived stem cells (hMADS)	Increased mRNA levels of *ATG5, ATG7,* and *ATG12* in AT in overweight/obese individuals Differentiated hMADS treated with a hormone-sensitive lipase inhibitor increased LC3 accumulation	Upregulated expression of genes involved in autophagosome formation	[42]

^a^The authors do not specify how many patients were recruited in total, however, based on figure legends in the results, the groups including patients with and without T2D consisted of 5 subjects per group for TEM; 7 subjects per group for autophagic activity, amount, and turnover of LC3; 17 subjects without T2D and 10 subjects with T2D for quantification of lipofuscin particles

^b^The specific number of persons per group, from whom AT biopsy was used to assess markers of autophagy was not specified

^c^*p62/SQSTM1* mRNA quantification involved adipocytes from 14 obese and 10 nonobese patients

It is important to emphasize that AT biopsy samples are highly heterogeneous, owing to the combination of numerous cell types. Besides adipocytes, AT also contains the stromal-vascular fraction, which includes obesity-associated inflammatory cells whose role in autophagy is related to immune function. Therefore, it is unclear, which cell type presented an upregulated autophagy profile in some of the previous reports. However, some discrepancies may exist, even between studies performed using the same cell type. For example, in contrast with the results reported by Ost et al. [38], Soussi et al. demonstrated that autophagic flux was greatly diminished in adipocytes freshly isolated from subcutaneous AT of obese patients compared with control adipocytes [45]. Moreover, decreased autophagic flux was inversely correlated with the amount of lipids accumulated in these adipocytes. Accordingly, this outcome was partially reversed after bariatric surgery, proportionally to the reduction of adipocyte size [45]. The mechanism of autophagy attenuation was shown to be dependent on death-associated protein kinase 2 (DAPK2), reported to be one of the most downregulated genes in the AT transcriptome in human morbid obesity [46].

To summarize, it could be expected that the impairment of systemic metabolism due to excessive food intake together with insufficient energy expenditure, which are inherently associated with obesity, lead to autophagy inhibition due to enhanced mTORC1 signaling. However, the evidence provided so far points to the fact that the impact of overnutrition on autophagy is much more complex. Autophagy can be either enhanced or inhibited, depending on changes in the global metabolism of a specific type of investigated organ, or tissue, as well as the experimental conditions or duration of the disease [36]. Most reports have provided evidence that autophagy in obesity is upregulated at the initiation stage of autophagosome biogenesis, which might serve as an autophagic response to proinflammatory conditions in AT [44]. Of note, the majority of these studies observed autophagy alterations based on the changes in the mRNA levels of only several genes (mostly *ATG5*, *ATG7*, and *LC3B*) [47]. Only a few of these alterations were confirmed also at the protein level. The exception was *LC3B*, whose protein levels were evaluated by Western blotting in almost all the studies.

### Type 2 diabetes

Two pathological conditions underlying T2D development, which are also associated with impaired autophagy, are insulin resistance and β-cell failure [48, 49]. Elevated glucose levels have been shown to disturb autophagy in vitro and in vivo, resulting in the aggravation of diabetes-related metabolic derangements in insulin-target tissues, such as skeletal muscle, which contribute to the worsening of insulin resistance [50, 51]. Accordingly, Møller et al. reported that the mRNA and protein levels of autophagy-related molecules were downregulated in the skeletal muscle biopsies of patients with T2D and severe insulin resistance [52]. Given that the studied patients were infused with insulin and glucose during biopsy sampling, it is difficult to conclude whether the observed effects reflect the disease state, treatment, or disease complications [52]. This limitation was overcome by Kruse et al., who showed that neither obesity nor T2D affected the expression of numerous autophagy-related genes at both mRNA and protein levels in muscle and AT biopsies, despite hyperglycemia (Table 2) [53]. Skeletal muscle biopsies were obtained under basal and insulin-stimulated states during euglycemia and hyperglycemia, and these experiments showed that physiological insulin concentrations reduced the levels of markers of autophagosome formation in an mTOR-independent manner. The impact of insulin in patients with T2D was no longer detectable under euglycemia but was restored under a hyperglycemic state. These results implied that the autophagic process may be adapted to hyperglycemic conditions in patients with T2D [53].

**Table 2:** Studies demonstrating autophagy alterations in patients with T2D

Cohort	Material	Autophagy markers	Interpretation	Reference
Controls without T2D (*n* = 12) Patients with T2D (*n* = 14)	Pancreatic islets	Higher density volume of autophagosomes in diabetic β-cells Decreased *LAMP2* and cathepsin B and D mRNA levels No changes in *BECN1* and *ULK1/ATG1* mRNA levels	Increased formation of autophagosomes in T2D β-cells	[54]
Controls without T2D (*n* = 30) Patients with T2D (*n* = 47)	Pancreas (*post-mortem*)	Increased levels of p62/SQSTM1 in patients with T2D with severe β-cell loss	Decreased lysosomal degradation	[55]
Healthy lean controls (*n* = 12) Obese individuals without T2D (*n* = 8) Obese patients with T2D (*n* = 10)	Skeletal muscle biopsy and subcutaneous AT	**Muscle:** *ULK1, BECN1, ATG5, ATG7, ATG12, LC3B, GABARAPL1, p62/SQSTM1, BNIP3,* and *BNIP3L* mRNA levels not affected Levels of ATG7, BNIP3 and p62/SQSTM1 proteins not affected Increased level of p62/SQSTM1 protein in response to insulin in patients with T2D under euglycemia but not during hyperglycemia Decreased level of LC3B-II and LC3B-II/I ratio in response to insulin in patients with T2D under hyperglycemia compared to euglycemia **AT: ***ATG5, ATG7, BECN1, BNIP3, LC3B,* and *p62/SQSTM1* mRNA levels not affected	Decreased formation of autophagosomes under physiological insulin concentrations Decreased lysosomal degradation under euglycemia suggests adaptation to hyperglycemia in patients with T2D	[53]
Controls without T2D (*n* = 109) Patients with T2D (*n* = 103)	Leukocytes	Increased level of BECN1 and LC3-II proteins	Increased formation of autophagosomes	[60]
Healthy controls (*n* = 7) Patients with T2D (*n* = 7)	Skeletal muscle biopsy	Downregulated *ATG14, GABARAPL1, RB1CC1/FIP200, WIPI1*, and *p62/SQSTM1* mRNA levels in diabetic patients with T2D Downregulated levels of LC3B-II, p62/SQSTM1 and ATG5 proteins in patients with T2D	Decreased formation of autophagosomes and lysosomal degradation	[52]
Controls without T2D (*n* = 10) Patients with T2D (*n* = 10)	Peripheral blood mononuclear cells	Downregulated *BECN1*, *LAMP2* mRNA levels in patients with T2D *LC3B* and *ATG5* mRNA levels were not affected in patients with T2D Decreased level of LC3B-II protein Increased level of p62/SQSTM1 protein Levels of ATG5 and ATG7 proteins were not affected in patients with T2D	Decreased autophagic flux	[59]
Controls without T2D (*n* = 12) Patients with T2D (*n* = 6)	Pancreatic islets	Decreased levels of LC3 and p62/SQSTM1 proteins in patients with T2D	Decreased level of autophagy	[58]
Cardiovascular complications				
Controls without T2D (*n* = 41) Patients with T2D (*n* = 45)	Endothelial cells	Increased levels of ATG7, p62/SQSTM1 and LAMP2A proteins in patients with T2D No changes in the level of the total LC3 and BECN1 proteins	Decreased lysosomal degradation	[62]
Non-ischemic controls without T2D (*n* = 14) Ischemic patients without T2D (*n* = 14) Ischemic patients with T2D (*n* = 15)	Heart tissue (right atrial appendage)	Increased level of LC3B-II protein in diabetic cardiomyocytes Increased level of BECN1 protein in nondiabetic ischemic and diabetic cardiomyocytes Decreased level of p62/SQSTM1 protein in diabetic cardiomyocytes Increased number of autophagosomes in diabetic cardiomyocytes	Increased autophagic flux	[65]
Nephropathy				
Healthy controls (living allograft donors) (*n* = 18) Patients with nonprogressive proteinuric states (*n* = 5) Patients in the early stage of diabetic nephropathy (*n* = 22)	Kidney biopsy	Induced mRNA expression of mTORC1 and its target genes in patients with progressive disease	Upstream inhibition of autophagy	[66]
Patients without T2D with different types of nephropathy (*n* = 14) Patients with diabetic nephropathy and massive proteinuria (*n* = 7) Patients with diabetic nephropathy and minimal proteinuria (*n* = 4)	Kidney biopsy	Increased level of p62/SQSTM1 protein in patients with diabetic nephropathy and massive proteinuria	Decreased lysosomal degradation	[67]
Healthy controls (*n* = 20) Patients with T2D (*n* = 70)	Serum	Decreased levels of BECN1 protein	Decreased autophagophore nucleation	[72]
Healthy controls (*n* = 18)^b^ Patients with T2D (*n* = 20)^c^ Patients with diabetic retinopathy (*n* = 24)^c^ Patients with diabetic nephropathy (*n* = 23)^b^	Peripheral blood mononuclear cells	Decreased level of ATG5 and LC3B proteins in all patients with T2D	Decreased formation of autophagosomes	[73]

^a^*n* = 9 for protein levels

For LC3B, sample sizes were:

^b^*n* = 19

^c^*n* = 18

In 2009, Liang et al. showed that β-cells obtained from patients with T2D exhibited a major accumulation of autophagic phagosomes. The expression of genes involved in the initial phases of autophagy was not affected in diabetic islets, however, a lower expression of lysosomal genes suggested the presence of alterations at later stages, which could disrupt autophagosome removal capacity. These findings were associated with β-cell dysfunction and failure [54]. These results were in line with the observations of Mizukami et al., who reported that p62/SQSTM1 protein levels were increased in pancreases from patients with T2D (obtained *post-mortem*), suggesting defective autolysosomal degradation, but only if severe β-cell loss was observed [55]. Further studies have shown that the structure of pancreatic islets is maintained by the autophagy process, which contributes to the survival of pancreatic β-cells, exerting protective effects on them and insulin-target tissues [56, 57]. For example, autophagy has been shown to prevent β-cell death during hypoxia caused by a rapid metabolic rate to supply insulin production. Immunofluorescence staining of pancreatic tissue from patients with T2D revealed decreased protein expression of LC3B and p62/SQSTM1, two key autophagic markers, which may be related to chronic hypoxic conditions in diabetic islets [58]. To further support the connection between autophagy impairment and the glycemic state of patients, the levels of autophagic markers and the extent of β-cell loss were reported to be negatively correlated with the levels of HbA1c [55, 58]. Decreased autophagic flux in patients with T2D may also be related to enhanced mTOR signaling and inflammation, as reported by Alizadeh, who evaluated the association between T2D and inflammation in peripheral blood mononuclear cells (PBMCs) [59]. In contrast, elevated levels of Beclin 1 (BECN1) and LC3B, suggesting enhanced autophagosome biogenesis, were positively correlated with the extent of oxidative and ER stress markers in leukocytes obtained from T2D patients. These findings point to a potential cell-dependent divergence that can occur when studying autophagic flux among patients with T2D [60].

### Diabetic complications

To date, it has been ascertained that aberrant regulation of autophagy, which occurs in patients with T2D, may be responsible for the occurrence and progression of diabetic complications (Table 2). Persistent hyperglycemia and insulin resistance trigger disruption of cellular metabolism, resulting in a decline in tissue and organ function. This further contributes to the development of diverse diabetic complications that affect the cardiovascular system, kidneys, nerves, and eyes, such as diabetic heart disease, diabetic kidney disease, diabetic nephropathy, diabetic peripheral neuropathy, and diabetic retinopathy (DR) [61, 62]. For example, patients with T2D have a higher risk of cardiovascular complications than healthy individuals. High blood glucose levels induce oxidative stress and chronic inflammation in endothelial cells, favoring endothelial injury with an augmented risk of atherosclerosis, myocardial infarction, stroke, and limb amputation [63]. Alterations in cardiac metabolism result in the inhibition of lysosomal degradation, leading to the accumulation of defective organelles in diabetic cardiomyocytes with consequent cardiac damage [64, 65]. The defective terminal phase of autophagy in endothelial cells isolated from patients with T2D was demonstrated by increased levels of p62/SQSTM1 and LAMP2A proteins. In parallel, an increased level of ATG7 protein suggested enhanced autophagosome formation; however, the level of BECN1, which is involved in the initiation of autophagosome biogenesis, was not significantly affected [66]. This report is in contrast to the findings of Munasinghe et al., who reported an increased number of autophagosomes and significantly elevated levels of LC3B-II and BECN1 proteins in diabetic cardiomyocytes. At the same time, the level of p62/SQSTM1 was reduced, implying stimulation rather than inhibition of the autophagic process [67].

Another chronic complication associated with diabetes is diabetic nephropathy, a pathological condition, in which the renal tissue is highly susceptible to irreversible damage due to prolonged high blood glucose and lipid levels, which favor microvascular injury and lead to oxidative stress, resulting in podocyte toxicity [68, 69]. Persistent hyperglycemia contributes to mTORC1 activation, which disrupts autophagosome formation in podocytes of mice and patients with T2D, resulting in exacerbated proteinuria [70]. Tagawa et al. showed that the level of p62/SQSTM1 protein was elevated in kidney biopsies from patients with diabetic nephropathy and massive proteinuria, suggesting defective lysosomal degradation [71]. The level of BECN1 in the serum of patients with diabetic kidney disease was reported to be decreased, which was inversely correlated with the severity of albuminuria, stage of nephropathy, and duration of diabetes [72]. Accordingly, lowered autophagosome biogenesis, reflected by decreased levels of ATG5 and LC3B proteins in patients with T2D, including those with nephropathy or retinopathy, has also been reported [73].

Altogether, some studies indicate that autophagosome formation is enhanced in response to metabolic stressors, such as insulin resistance and hyperglycemia, potentially serving as a compensatory mechanism to remove damaged organelles and proteins. Conversely, other studies suggest that chronic hyperglycemia and dysregulated insulin signaling impair autophagic flux, leading to the accumulation of dysfunctional cellular components and contributing to the pathogenesis of insulin resistance and β-cell dysfunction. These equivocal outcomes underscore the complex role of autophagy in metabolic dysregulation implicated in the pathogenesis of T2D.

### Metabolic dysfunction-associated steatotic liver disease

The prevalence of MASLD is highly associated with the presence of both obesity and T2D. In contrast to obesity (or adipocytes, to be more specific), where the current evidence implies enhanced autophagy, data gathered from MASLD patients suggest that autophagy in the liver is mostly inhibited [36]. In 2014, Fukuo et al. demonstrated by TEM that the number of autophagic vesicles in liver samples obtained from MASLD patients was three times higher than that observed in histopathologically normal livers [74]. On the other hand, TEM analysis of liver sinusoidal endothelial cells (LSECs) from liver biopsies carried out by Hammoutene et al. revealed that autophagic vacuoles are notably smaller and less numerous in patients with metabolic dysfunction-associated steatohepatitis (MASH) compared to those with simple steatosis or no liver abnormalities, implying defects in autophagic processes (Table 3). Furthermore, in vitro mechanistic experiments performed on immortalized liver endothelial cells suggest that defective autophagy may promote the generation of proinflammatory factors, such as tumor necrosis factor α (TNF-α) and interleukin 6 (IL-6), leading to endothelial inflammation and cell death [75]. Additional analysis based on immunohistochemical staining performed by Fukuo et al. revealed that in 15 out of 22 MASLD patients, p62/SQSTM1 levels were significantly elevated compared to the complete absence of this protein in the liver samples of the control group [74]. Significantly decreased expression of lysosomal enzymes (cathepsin B, D, and L) in the livers of MASLD patients supports the hypothesis of impaired autophagy, as an accumulation of p62/SQSTM1 indicates dysfunctional lysosomal degradation of autophagic cargo. Moreover, p62/SQSTM1 protein levels were significantly correlated with alanine aminotransferase levels, lobular inflammation, and NAS scores (especially scores higher than 5, indicating the presence of MASH) [74]. Other authors have also observed a significant accumulation of p62/SQSTM1 protein in the livers of patients with MASLD compared to individuals with histologically normal livers, with a more pronounced increase in p62/SQSTM1 protein aggregation observed in MASH compared to simple steatosis [76, 77]. Accordingly, Wang et al. reported increased p62/SQSTM1 levels in serum and liver tissue obtained from MASH patients, as well as in liver tissue samples from patients with simple steatosis [78]. The blockade of the autophagic flux was also supported by an increased LC3-II/LC3-I ratio, where LC3-II serves as a recognition site for p62/SQSTM1 [76]. An increased LC3-II/LC3-I ratio was also observed in liver samples from MASLD patients with prediabetes, nonetheless, the level of *p62/SQSTM1* protein was not affected in the studied cohort, which complicates the interpretation of these results [79]. No changes in p62/SQSTM1 levels along with an increased LC3A/B-II ratio were also reported by Lee et al., however, the evidence can be considered weak due to the extremely low sample size [80].

**Table 3:** Studies demonstrating autophagy alterations in MASLD patients

Cohort	Material	Autophagy markers	Interpretation	Reference
Controls with liver metastatic tumors (normal tissue excised from areas surrounding a tumor) (*n* = 14) MASLD patients (*n* = 22)	Liver biopsy	Increased number of autophagic vesicles Increased level of p62/SQSTM1 protein	Increased autophagosome formation and decreased lysosomal degradation	[74]
Controls with histologically normal liver (*n* = 34) MASLD patients without T2D (*n* = 49)	Liver biopsy	Lower *BECN1* mRNA level in patients with simple steatosis compared to patients with healthy liver and MASH patients, but no differences at the protein level Gradually rising p62/SQSTM1 protein level from normal to MASH livers Increased LC3 II/I protein ratio in MASLD	Downregulated expression of a gene involved in autophagosome formation (only in simple steatosis) and decreased lysosomal degradation	[76]
Controls with histologically normal liver (*n* = 19) Patients with simple steatosis (*n* = 10) MASH patients (*n* = 9) MASH patients without steatosis (*n* = 7)	Liver biopsy	Up-regulated autophagy-related genes in MASLD	Upregulated expression of genes involved in autophagic processes	[84]
Controls with histologically normal liver (*n* = 4) Patients with simple steatosis (*n* = 2) MASH patients (*n* = 3)	Liver biopsy	Increased levels of ATG16L1 protein and LC3A/B-II protein ratio in MASH No changes in p62/SQSTM1 protein	Increased autophagosome formation	[80]
Healthy controls (*n* = 66) Non-MASH patients (*n* = 59) MASH patients (*n* = 74)	Liver biopsy Serum	Increased p62/SQSTM1 protein level in hepatic tissue of MASH patients Increased level of p62/SQSTM1 protein in the serum of MASLD individuals	Decreased lysosomal degradation	[78]
Obese individuals with normal glycemia (*n* = 38) Obese MAFLD patients with impaired glucose tolerance (*n* = 32)^a^ Obese MAFLD patients with T2D (*n* = 33)^b^	Liver biopsy	No changes in *BECN1, ATG5,* and *ATG7* mRNA levels Increased LC3B II/I protein ratio in a prediabetic state No changes in p62/SQSTM1 protein level	Expression of genes involved in autophagosome formation not affected Increased level of a marker of autophagosome formation only in a prediabetic state	[79]
Controls with liver metastatic tumors (normal tissue excised from areas surrounding the tumor) (*n* = 5) MASLD patients (*n* = 31)	Liver biopsy	Increased number of autophagic vesicles with the morphology of autolysosomes Increased p62/SQSTM1 protein level	Increased formation of autolysosomes and decreased lysosomal degradation	[77]
Controls with histologically normal liver (*n* = 5) Patients with simple steatosis (*n* = 6) MASH patients (*n* = 12)	Liver biopsy Sinusoidal endothelial cells isolated from the liver (LSECs)	Decreased number and size of autophagic vacuoles in MASH patients	Decreased formation of autophagosomes	[75]
Controls with healthy liver (*n* = 13) Patients with simple steatosis (*n* = 34) Borderline MASH patients (*n* = 27) Definite MASH patients (*n* = 56)	Liver biopsy	Decreased levels of ULK1, p-ULK^s555^, BECN1, ATG5, p62/SQSTM1, and BNIP3 proteins in all MASLD subjects compared to controls with healthy liver Levels of ATG7, LC3-II proteins and LC3-II/I protein ratio were not significantly affected	Downregulated initiation of autophagy and autophagosome formation	[81]
Lean controls (*n* = 6) Morbidly obese patients: Non-steatotic (*n* = 11) With simple steatosis (*n* = 29) MASH (*n* = 32)	Liver biopsy	Higher mRNA and protein levels of *ATG7* in MASH patients	Upregulated autophagosome formation	[82]

^a^2 patients had histologically normal liver

^b^1 patient had histologically normal liver

BECN1 is implicated in the initiation process of the formation of autophagic vesicles. According to González-Rodríguez et al., a decrease in hepatic mRNA levels of *BECN1* was observed in patients with simple steatosis compared to patients with histologically normal liver or patients with MASH [76]. In line with these findings, decreased levels of BECN1 as well as other proteins, i.e., the Unc-51-like autophagy-activating kinase 1 (ULK1), p-ULKs555, ATG5, p62/SQSTM1, and BCL2 Interacting Protein 3 (BNIP3), were reported in liver samples from MASLD subjects compared to controls with healthy liver, which implies the impairment of initiation of autophagy and autophagosome formation [81]. On the other hand, González-Rodríguez et al. found no significant changes in BECN1 protein expression in liver tissue of MASLD patients, despite the aforementioned decrease in its mRNA levels [76]. Interestingly, Ezquerro et al. did not observe any changes in *BECN1* transcript levels in the liver tissue of MASLD patients, regardless of their glycemic status (distinction of *BECN1* levels between simple steatosis and MASH within the MASLD group was not performed) [79].

ATG5, ATG7, and LC3 proteins are involved in the maturation of the autophagosome. It has been reported that ATG5 transcript levels were not altered in patients with morbid obesity and MASLD, who exhibited different levels of insulin resistance [79]. In line with these results, both transcript and protein levels of ATG7, as well as protein levels of LC3-II and the LC3-II/I ratio, did not differ between controls and MASLD patients [81]. On the other hand, higher hepatic mRNA and protein expression of *ATG7* was reported in the MASH group, especially when mild lobular inflammation was present, within the cohort of morbidly obese women. However, the differences were revealed only when comparing MASH to non-MASH groups, but not MASLD vs. non-MASLD [82]. This finding requires further investigation and confirmation since recently it has been shown that loss-of-function mutations in the *ATG7* gene increased the risk of developing severe liver diseases among MASLD patients [83]. On the other hand, the gene set enrichment analysis performed by Lake et al. showed that the set of genes associated with autophagy was enriched among upregulated genes in liver biopsy samples obtained from patients with simple steatosis and MASH [84]. The authors suggest that the upregulation of the autophagy gene set may reflect an adaptation to hepatic lipotoxicity or ER-stress response [84], as also shown by González-Rodríguez et al. [76].

Most of the studies reported autophagy alterations at the protein level through the evaluation of LC3B, p62/SQSTM1, or both. Only a few studies investigated the mRNA levels of genes involved in the initiation of autophagosome formation, such as *BECN1*, *ATG5,* or *ATG7*. To conclude, the available evidence from patients supports the role of inhibition of the autophagic process in the development and progression of MASLD, however, more evidence regarding the stage of autophagy impairment is needed to better understand the pathophysiology of this disease.

## Autophagy modulators

**Fig. 2:**
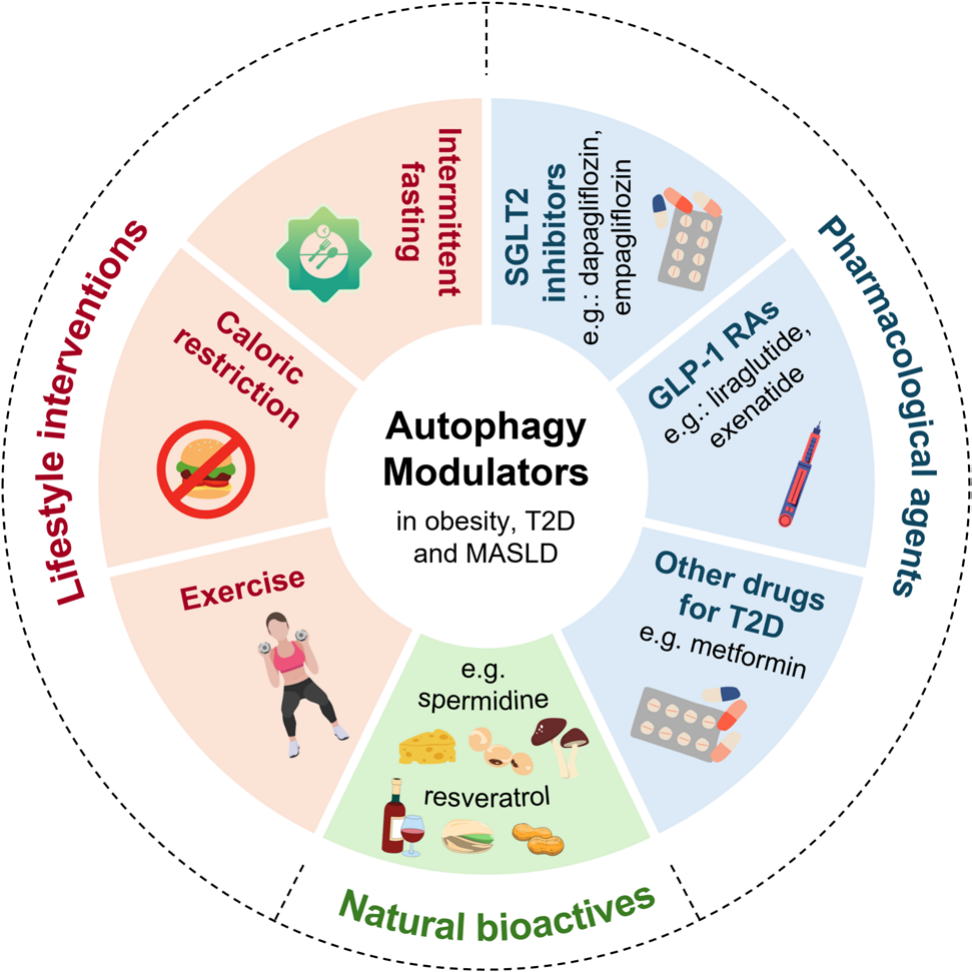
Autophagy-modulating strategies in obesity, type 2 diabetes (T2D) and metabolic dysfunction-associated steatotic liver disease (MASLD)

Autophagy-modulating strategies (Fig. 2) have gained significant attention as potential candidate therapies for numerous diseases including metabolic conditions such as obesity, MASLD, and T2D [14, 85, 86]. These strategies aim to restore the proper function of the autophagy process, which is critical for the maintenance of cellular homeostasis by degrading and recycling damaged organelles and proteins. Lifestyle interventions such as caloric restriction, intermittent fasting, and exercise have been shown to stimulate autophagy and improve metabolic health [87, 88]. Weight loss and exercise are known to be effective in the management of obesity, MASLD, and T2D [36, 89]. Even though controlled human studies assessing the impact of lifestyle interventions on autophagy markers among patients with obesity, MASLD or T2D are limited [87], Nunez et al. have shown that the elevated number of autophagosomes in the subcutaneous AT of patients with obesity became undetectable one year after bariatric surgery [40].

Besides lifestyle-based interventions, autophagy impairments in metabolic diseases could also be targeted with the use of pharmacological agents, for example, caloric restriction mimetics, which imitate caloric restriction-like benefits without following a dietary restriction [90, 91], AMPK activators, mTOR inhibitors or compounds restoring proper lysosomal function. Among drugs commonly used in clinical practice, metformin is a known autophagy modulator that regulates the AMPK pathway [92, 93]. According to the study by Abad-Jimenez et al., obese patients with T2D treated with metformin exhibited an improved inflammatory and redox status accompanied by the attenuation of inflammasome complex and autophagy in the visceral AT compared to metabolically healthy obese subjects. Markers of autophagy inhibition included decreased protein levels of ATG5, BECN1, and elevated levels of p62/SQSTM1 [94].

Inhibitors of sodium-glucose cotransporter 2 (SGLT2) constitute another class of drugs used to treat T2D, which apart from reducing blood glucose levels and body mass, enhance autophagy and lysosomal degradation [95]. To investigate the impact of dapagliflozin, one of the SGLT2 inhibitors, on the liver, Furuya et al. performed in vivo experiments followed by a small clinical study among hospitalized patients with T2D (*n* = 12) [96]. In an animal model of obesity and T2D (KK-Ay mouse strain), dapagliflozin increased the LC3-II/LC3-I ratio and the pool of several amino acids (valine, leucine, tryptophan, and tyrosine) in the liver suggesting the occurrence of enhanced proteolysis. Accordingly, plasma valine and leucine levels were significantly elevated among T2D patients, who received 5 mg of dapagliflozin per day together with other antidiabetic drugs compared to those who received antidiabetic drugs excluding dapagliflozin [96]. Also, empagliflozin, another SGLT-2 inhibitor, has demonstrated beneficial effects against hepatic steatosis under both in vitro and in vivo conditions [97]. In addition to its glucose-lowering effect, this drug alleviated hepatic steatosis through activation of the AMPK-TET2 pathway in hepatocytes, while in liver macrophages it potentiated autophagy in an AMPK/mTOR-dependent manner to attenuate the inflammatory response [97].

GLP-1 receptor agonists (GLP-1 RAs), primarily known for their efficacy in the treatment of T2D and obesity, have recently gained attention for their potential effects on autophagy [98]. One of the GLP-1 RAs, liraglutide, was found to reduce lipid accumulation in the liver by restoring autophagic flux via the AMPK/mTORC1 and transcription factor EB (TFEB)-mediated lysosomal autophagic pathways in both in vitro and in vivo experiments [99–101]. TFEB is recognized as a central transcriptional regulator of autophagy, a master regulator of lysosomal biogenesis, and a key regulator of lipid metabolism [102], which makes it a promising target for therapeutic intervention in metabolic diseases. Exenatide is another GLP-1 RA that has been studied for its autophagy-modulating effects. Shao et al. reported that exenatide targets the NLRP3 inflammasome via the autophagy/mitophagy pathway, delaying the progression of MASLD in mice [103]. Nevertheless, these findings are limited solely to in vitro and in vivo animal experiments.

Additionally, natural compounds like resveratrol and spermidine have demonstrated their potential in vivo in modulating autophagy and improving metabolic outcomes [104–106]. However, while resveratrol has shown potential metabolic benefits in in vitro and animal studies, evidence from human clinical trials remains limited and inconclusive. Most human studies have demonstrated only modest metabolic improvements, so many questions about its bioavailability, optimal dosing, and overall efficacy remain unanswered [107, 108]. Concerning evidence of autophagy modulation in humans, a 30-day supplementation with resveratrol (150 mg per day) was reported to induce TFEB expression in the subcutaneous AT of healthy obese men [109]. Another autophagy inducer, spermidine, has shown promising effects on metabolic health, including reducing weight gain, obesity-related complications, and improving insulin resistance in both humans and mice [110]. In particular, studies have demonstrated that spermidine intake negatively correlates with obesity-associated parameters and enhances gut barrier function, indicating its potential as a therapy for obesity and its complications [111]. So far, clinical evidence that spermidine supplementation may have beneficial effects through the regulation of autophagy in patients with obesity, T2D, and MASLD is lacking [112].

Therapeutic approaches targeting autophagy may hold promise for mitigating the progression of metabolic diseases and improving overall health. Nevertheless, further research is needed to fully understand and validate their potential for enhancing metabolic health in clinical settings through autophagy modulation.

## Conclusions

Autophagy is a fundamental cellular process that plays a pivotal role in the maintenance of metabolic homeostasis. In metabolic diseases such as obesity, T2D, and MASLD, dysregulation of autophagy has emerged as a key contributing factor. Autophagy regulates lipid metabolism, insulin sensitivity, and glucose homeostasis, affecting the development and progression of these metabolic disorders. In this review, we gathered evidence of autophagy impairments from human studies, which clearly shows that autophagy alterations may differ depending on the type of tissue or cells. Due to methodological limitations, it is difficult to conclude whether the observed changes in autophagic flux are a cause or a consequence of the metabolic diseases we have discussed. Understanding the intricate interplay between autophagy and different metabolic pathways is crucial for developing effective therapies for metabolic diseases, nevertheless, further research is needed to identify optimal targets for potential interventions.

## Data Availability

Not applicable.
