# Correction: Autophagy alterations in obesity, type 2 diabetes, and metabolic dysfunction-associated steatotic liver disease: the evidence from human studies

**DOI:** 10.1007/s11739-024-03822-1

**Published:** 2024-11-26

**Authors:** Patrycja Jakubek, Barbara Pakula, Martin Rossmeisl, Paolo Pinton, Alessandro Rimessi, Mariusz Roman Wieckowski

**Affiliations:** 1 Laboratory of Mitochondrial Biology and Metabolism, Nencki Institute of Experimental Biology, Polish Academy of Sciences, 3 Pasteur St., 02-093 Warsaw, Poland; 2 Laboratory of Adipose Tissue Biology, Institute of Physiology of the Czech Academy of Sciences, Prague, Czech Republic; 3 Department of Medical Sciences, Section of Experimental Medicine, Laboratory for Technologies of Advanced Therapies, University of Ferrara, Ferrara, Italy; 4 Center of Research for Innovative Therapies in Cystic Fibrosis, University of Ferrara, 44121 Ferrara, Italy

**Correction: Internal and Emergency Medicine (2024) 19:1473–1491** 10.1007/s11739-024-03700-w

In the Acknowledgements section of this article the grant number was incorrectly given as 22-04100L and should have been 23-04100L.

The Original article has been corrected.

